# In Vivo Degradation Behavior of AZ91 Magnesium Alloy: Comprehensive Microstructural and Crystallographic Characterization by TEM and NBED

**DOI:** 10.3390/ma18071500

**Published:** 2025-03-27

**Authors:** Zhichao Liu, Honglei Yue, Jianhua Zhu, Jianmin Han

**Affiliations:** 1 Department of Dental Materials, Peking University School and Hospital of Stomatology, Beijing 100081, China; 2 National Center for Stomatology & National Clinical Research Center for Oral Diseases & National Engineering Research Center of Oral Biomaterials and Digital Medical Devices & Beijing Key Laboratory of Digital Stomatology & NHC Key Laboratory of Digital Stomatology & NMPA Key Laboratory for Dental Materials, Beijing 100081, China; 3 Department of Oral and Maxillofacial Surgery, Peking University School and Hospital of Stomatology, Beijing 100081, China; 4 Central Laboratory, Peking University School and Hospital of Stomatology, Beijing 100081, China; 5 School of Stomatology, Tianjin Medical University, Tianjin 300070, China

**Keywords:** AZ91, corrosion mechanism, in vivo, TEM, nanobeam electron diffraction (NBED)

## Abstract

Magnesium alloys have attracted significant attention in recent years as biodegradable metals. However, their degradation mechanisms in vivo remain insufficiently understood. The present work investigates the degradation mechanism of AZ91 magnesium alloy in a critical-size rat defect model over an 8-week period in vivo, employing advanced characterization techniques such as transmission electron microscopy (TEM) and nanobeam electron diffraction (NBED). The degradation layer is observed to consist of three distinct sub-layers: a dense and compact poor crystallinity layer (PCL) layer primarily composed of calcium phosphate, a loose and porous amorphous layer (AL) of magnesium/calcium phosphate, and a hybrid layer (HL)layer containing degradation channels and composed of magnesium/calcium phosphate, layered double hydroxide (LDH), and magnesium hydroxide. The corrosion resistance of AZ91 is enhanced by the presence of the compact PCL layer, the uniform distribution of the Mg_17_Al_12_ phase, and the formation of impervious LDH at the corrosion interface. The degradation is primarily driven by micro-galvanic corrosion, which is influenced by the interaction between the Mg matrix and the Mg_17_Al_12_ phase. These findings provide critical insights into the stable degradation mechanism of Mg-Al alloys in vivo, advancing the development of biodegradable magnesium-based implants.

## 1. Introduction

Biodegradable magnesium (Mg) and its alloys are emerging as promising candidates for use as biomedical implant materials, owing to their biodegradability, non-toxicity, and excellent physical and mechanical properties [1,2,3,4]. The Mg alloy is becoming one of the most promising biomaterials, especially in orthopedics [5], stomatology, and vascular intervention. These fields require appropriate design of the composition, processing, microstructure regulation, and surface modification of the alloys that are used to adapt to clinical demands. Nevertheless, the unmanageable and unpredictable degradation characteristics of Mg alloy in physiological environments hinder its extensive application in clinics. The degradation characteristics of the Mg alloy due to its low electrochemical potential and its leaky protective film are involved in hydrogen evolution, element/ion exchange, local pH increase, toxicity, allergization, and undesirable biological reactions [6]. The degradation layer that forms on an implanted Mg device interacts directly with the surrounding physiological environment, exerting a pivotal influence on the degradation behavior of the device and the ensuing biological responses. The degradation layer may retard the degradation by slowing down substance transport and influence protein adsorption and cell attachment by changing the surface chemistry and morphology [7]. According to previous reports, the degradation products of Mg alloy in vivo generally contain magnesium hydrates, various types of (Ca/Mg)-phosphate, and Mg-carbonates [8], and these compounds have a significant impact on the biocompatibility of the device [9,10,11]. In addition, the degradation products formed on rare earth Mg alloys are highly detrimental to cells [12]. Thus, it is imperative to elucidate the specific characteristics, including the structural composition, of the degradation layer. Particular attention should be directed towards understanding the dynamic transformation of various ions between the alloy and bodily fluids at the degradation interface.

Numerous studies have been conducted to experimentally investigate the in vitro corrosion behavior and degradation products of Mg alloys. However, there exists a limited correlation between in vitro and in vivo corrosion of Mg alloys due to the oversimplification of in vitro studies. The degradation behavior of Mg alloys is notably complex when they are implanted within an organism. In vivo, a multitude of inorganic ions (e.g., Na^+^, K^+^, Ca^2+^, Mg^2+^, HCO^3+^, Cl^−^, HPO_4_^2−^, and SO_4_^2−^), organic components (such as proteins and amino acids), and buffer systems surrounding the implant profoundly influence the Mg alloy degradation. Furthermore, the synergistic and mutual effects of these factors render the simulation of metallic corrosion in vitro considerably challenging [13]. Hence, in vivo experiments are considerably more reliable.

While there is a significant amount of research on the in vivo degradation of magnesium alloys, there has been relatively limited research on the degradation layer. In these studies, the surface degradation products were determined via different techniques, such as scanning electron microscopy/energy-dispersive X-ray (SEM/EDX) [14], Fourier-transform infrared spectroscopy (FTIR) [15], X-ray photoelectron spectroscopy (XPS) [16], X-ray diffraction (XRD) [15], Auger electron spectroscopy (AES) [17], and transmission electronic microscopy (TEM) [18,19]. Among these techniques, TEM offers a better resolution than the others and is suitable for the nanoscale characterization of the microstructure and chemistry of degradation layers. In the realm of material phase analysis using TEM, the preferred technique frequently centers on the utilization of selected area electron diffraction (SAED). SAED necessitates the precise selection of a specific area aperture to delineate the region designated for electron diffraction analysis. Due to mechanical constraints, the minimum attainable diameter for the selected area aperture usually approximates 10 μm, which is usually accompanied by the associated objective lens magnification being set at 100×. Consequently, the smallest observable field of view achievable through the selected area aperture can reasonably be equated with the diameter of the sample, represented as D = 10 μm/100 = 0.1 μm. In this context, the dimensions of the selected area aperture and the objective lens magnification collectively impose restrictions on the smallest analyzable region. To overcome this issue, nanobeam electron diffraction has been developed [20]. Using this method, a lateral resolution of 0.01 μm or finer can be attained, and the resolution is primarily constrained by the dimensions of the incident electron beam [21].

The most widely used Mg-Al-Zn (AZ series) alloys offer an ultra-tensile strength up to ~400MPa [22]. The microstructure of AZ series alloy is comparable to a composite composed of a matrix of Mg reinforced by small islands of Mg_17_Al_12_. The phase (Mg_17_Al_12_) was usually introduced to improve the mechanical properties. While extensive research has been conducted on the degradation behavior of Mg alloys over the past few years, there remains a limited understanding of the intricate degradation mechanisms associated with intermetallic particles. For example, the AZ series Mg alloy has been widely investigated as a degraded biomaterial in vitro [23,24,25,26] and in vivo [9,10,27]. Microscopic examination of the in vivo degradation of Mg_17_Al_12_ has not yet been carried out. Among the AZ series of alloys, AZ91 contains a higher amount of aluminum (Al), which typically leads to a more abundant and evenly distributed Mg_17_Al_12_ phase. This phase is responsible for the alloy’s improved strength and enhanced corrosion resistance [13,28,29]. Hence, in the present investigation, a sample of AZ91 alloy featuring two distinct phases, namely the Mg matrix and Mg_17_Al_12_ phases, was prepared to study the degradation behavior of AZ91 and elucidate the correlation between the Mg matrix and β-Mg_17_Al_12_.

Typically, in the context of the in vivo degradation of Mg alloy implants, the degradation rate tends to stabilize following an initial phase of rapid degradation, which is primarily attributed to the formation of a protective layer [30]. Subsequently, the Mg alloy continues to degrade gradually as the interface between the Mg alloy and the protective layer undergoes transformations, culminating in the complete degradation of the Mg alloy. This sustained stable state endures for a relatively extended period, facilitating a more comprehensive opportunity for observation and analysis. In this research, we adopted a critical-size rat calvarial defect as the in vivo model for our study. Based on preliminary experiments, we observed that the test samples, characterized by a stabilized degradation layer, could be obtained after eight weeks of implantation. We conducted a range of multiscale analyses, which included the use of scanning electron microscopy with energy-dispersive X-ray spectroscopy (SEM-EDX), scanning transmission electron microscopy (STEM), high-resolution transmission electron microscopy (HRTEM), and nanobeam electron diffraction (NBED), to elucidate the structure and composition of the degradation layer. This study offers a comprehensive understanding of the degradation processes and mechanisms of Mg alloys containing Mg_17_Al_12_ at the nanometer scale in vivo.

## 2. Materials and Methods

### 2.1. Material Preparation and Mechanical Testing

**Figure 1:**
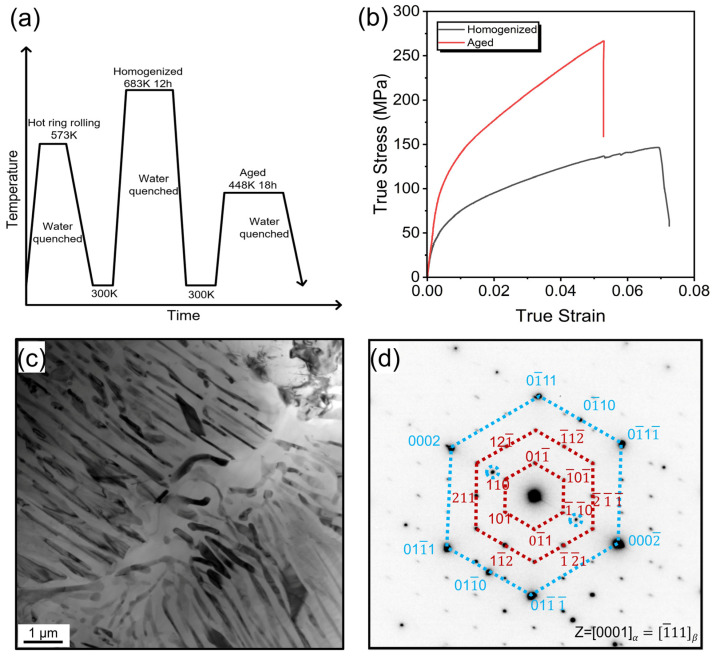
(**a**) Schematic processing route for the present AZ91. (**b**) True stress–strain curves of homogenized and aged sample. (**c**) TEM bright-field image. (**d**) SAED pattern corresponding to (**c**).

The AZ91 alloy, with the specific chemical compositions listed in Table 1, was prepared under an inert atmosphere of SF_6_ and CO_2_ cover gas in an induction furnace, and the subsequent processing route is illustrated in Figure 1a. First, the ingot was hot ring rolled (HRR) at 573 K. The detailed hot ring rolling operation principles have been described in [31]. After being HRR, the sample was homogenized at 683 K for 12 h, and then aged at 448 K for 18 h. To investigate the mechanical behavior of the present alloy, flat, dog-bone-shaped tensile samples with a gauge length of 10 mm were analyzed out at room temperature by an Instron 5966 universal testing machine (Instron, Boston, MA, USA)with a strain rate of 10^−3^ s^−1^. The loading direction was parallel to the rolling direction.

### 2.2. Implantation In Vivo

The membranes that were 5 mm in diameter and 0.11 mm in thickness were machined from the aged AZ91. The roughness *Ra* of the membrane was about 0.14 μm. Prior to implantation, the membranes were subjected to ultrasonic cleaning in acetone, ethanol, and distilled water, and sterilized with ultraviolet irradiation for 30 min. The membranes were inserted into the critical-size calvarial defect of five 8-week-old Sprague Dawley (SD) rats. Detailed surgical procedure can be seen in [32]. After an 8 week healing periods, five SD rats were euthanized, and the implant specimens, with the surrounding bone tissues, were harvested. The samples containing residual membranes and tissues were fixed in 90% ethanol solution. One of the samples was selected for degradation layer characterization. The research protocol was approved by the Animal Care and Use Committee of Peking University (Approval No. LA2020514). All animal experiments were conducted in accordance with the relevant ethical guidelines and regulations, and efforts were made to minimize animal suffering and reduce the number of animals used.

### 2.3. Degradation Layer Characterization

The selected sample was dehydrated in alcohol solutions with increasing concentrations (50 vol%, 75 vol%, 90 vol%, and 99 vol%) and then embedded in auto-polymerizing methyl methacrylate resin. The sample was sectioned using a diamond saw to expose the implant and bone, which was followed by further cutting with a Leica RM 2255 automated microtome (Leica, Wetzlar, Gemany) to obtain a smooth and fresh surface. The samples for scanning electron microscopy (SEM) examination were carbon-coated using a JEOL JFC-1600 auto fine coater (JEOL, Tokyo, Japan). The surface morphology was examined via FEI QUANTA 200F SEM (FEI, Hillsboro, OR, USA) in the backscattered electron (BSE) mode. The composition of degradation products from the surface to the inner region of the material was analyzed via EDAX octane elite energy dispersive X-ray spectrometry (EDX).

The area of specific interest, showing significant changes in element content as identified from the SEM and EDX analysis of the surface regions, was extracted using a JEOL JIB-4700F FIB-SEM (JEOL, Tokyo, Japan). This area was coated with a protective platinum (Pt) film and subsequently milled to produce an approximately 200 nm thick lamella with a depth of about 2–3 μm via a standard lift-out TEM sample preparation routine. The FIB gallium (Ga) ion beam was operated at an accelerating voltage of 5 kV to thin the FIB foil to a near 50 nm thickness.

The crystal structure and elemental composition of the TEM samples were characterized via Cs-corrected and mono-chromated FEI Titan G2 60–300 KV (FEI, Hillsboro, OR, USA), TEM, and bright-field (BF) images and STEM Z-contrast images at 300 kV acceleration voltages were acquired. Phase information was obtained from HRTEM and NBED. The NBED data were collected and recorded sequentially along scanning lines in STEM mode with the incoming beam parallel to the growth plane. The processing speed was set to its lowest value of 0.1 s. The electron beam was accelerated at a voltage of 200 kV. The current was less than 0.5 nA. Diffraction patterns were recorded on a 2 k × 2 k GATAN ultra-scan CCD camera with typical acquisition times of 1 s per pattern. Condenser apertures with diameters of 5 μm and 10 μm were used. These parameters were selected based on preliminary tests to ensure they were below the threshold for inducing significant beam damage. A typical series of 200 diffraction patterns lasted about 4 min. During the test, no significant structural changes were observed under the optimized conditions.

## 3. Results

### 3.1. Mechanical Properties and Microstructures of AZ91 Alloy

Figure 1b depicts the true stress–strain curves of the aged AZ91 in comparison to its homogenized counterpart. The yield stress and ultimate tensile strength of the aged alloy were measured at 130 MPa and 261 MPa, respectively, with a fracture elongation of 5.2%. The AZ91 alloy showed better mechanical properties than the AZ31 alloy [33], Mg-Ca alloy [34], and Mg-Ag alloy [35], which signifies its excellent supportive performance in vivo. The typical microstructure of AZ91 alloy is presented in Figure 1c,d, where a large amount of lamellar precipitates can be observed within the Mg matrix. The selected area electron diffraction (SAED) results confirm that the primary precipitates in the aged samples were Mg_17_Al_12_.

### 3.2. Degradation Layer Analysis via SEM and EDX

**Figure 2:**
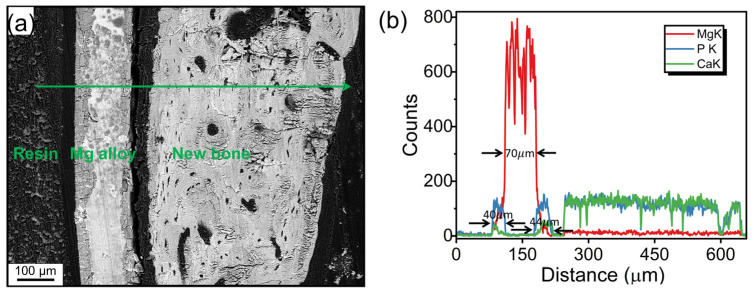
Cross-sectional morphology acquired by SEM with a BSE detector (**a**) and line (green) scan element distribution collected by EDS of the AZ91 alloy implanted in vivo for eight weeks (**b**).

The cross-sectional morphology, acquired by the SEM with a BSE detector, of the membrane implant that was placed in vivo for 8 weeks is shown in Figure 2a. The magnesium alloy exhibits nearly symmetrical degradation on both sides. Simultaneously, two clearly evident, relatively uniform degradation corrosion layers were formed, which created a sandwich-like structure with the non-degraded portion of the membrane. A substantial area of bone tissue formation is observed on the right side. When looking at the line (green) scan element distribution collected by the EDS (see Figure 2b), a well-defined three-layer structure can be seen. The residual undegraded membrane was about 70 μm thick. And the degradation layer with Ca and P enrichment on each side was about 40–44 μm thick. An elemental interdiffusion between the membrane and adjacent tissue can be inferred due to the continuous change in element content at the interface.

### 3.3. Degradation Layer Analysis via TEM and STEM

**Figure 3:**
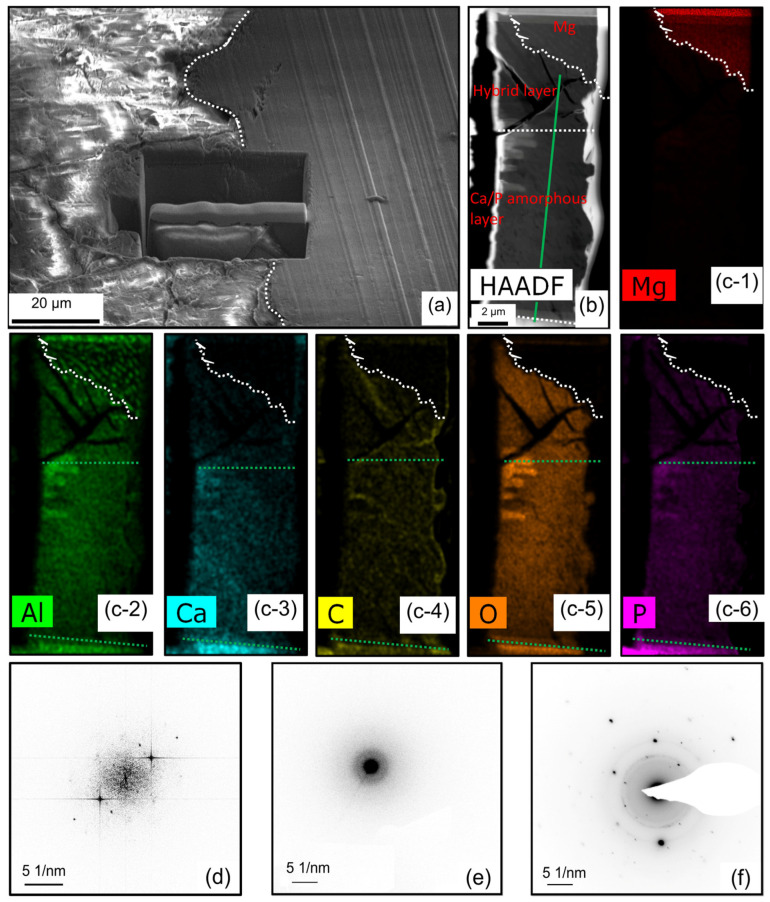
(**a**) A TEM specimen was lifted out from the corrosion interface. (**b**) The STEM-HAADF image. (**c-1**–**c-6**) Elemental distribution map of Mg, Al, Ca, C, O and P respectively corresponding to (**b**). The white dashed line corresponds to the corrosion front, while the green dashed lines denote the demarcations between distinct layers. The solid lines represent the path of NBED. (**d**), (**e**), and (**f**) are representative NBED patterns of the hybrid layer, Ca/P amorphous layer, and poor crystallinity layer in (**b**), respectively.

**Figure 4:**
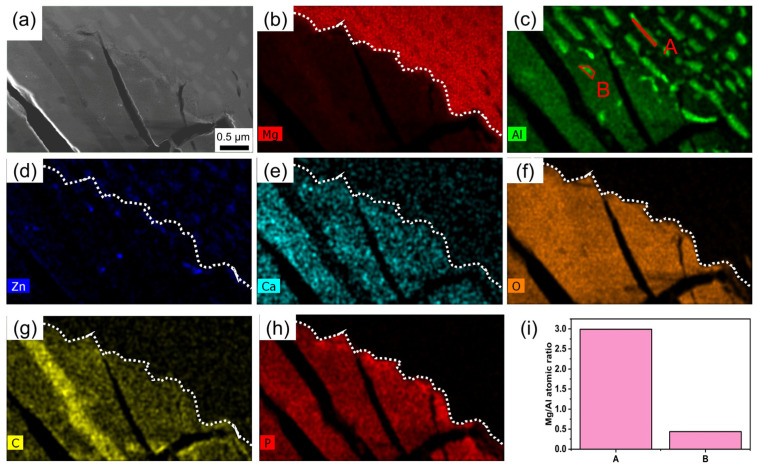
(**a**) The corrosion interface at a close proximity, visualized by the STEM-HAADF. (**b**–**h**) Elemental distribution map corresponding to (**a**). (**i**) Mg/Al atomic ratio of area A and B in (**c**).

**Figure 5:**
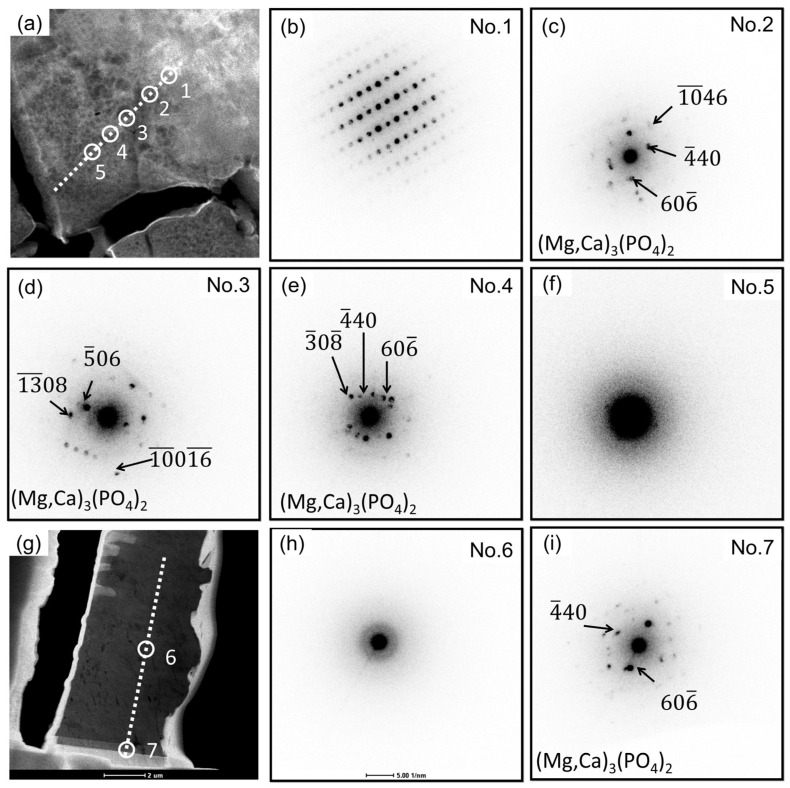
Representative nano-beam electron diffraction (NBED) patterns of HL, AL, and PCL layer. (**a**) The white dash line represents the continuous region of NBED.in HL. (**b**–**f**) The representative diffraction patterns in (**a**). (**g**) The white dash line represents the continuous region of NBED.in AL and PCL layer. (**h**) The representative diffraction patterns of AL layer. (**i**) The representative diffraction patterns of PCL layer.

**Figure 6:**
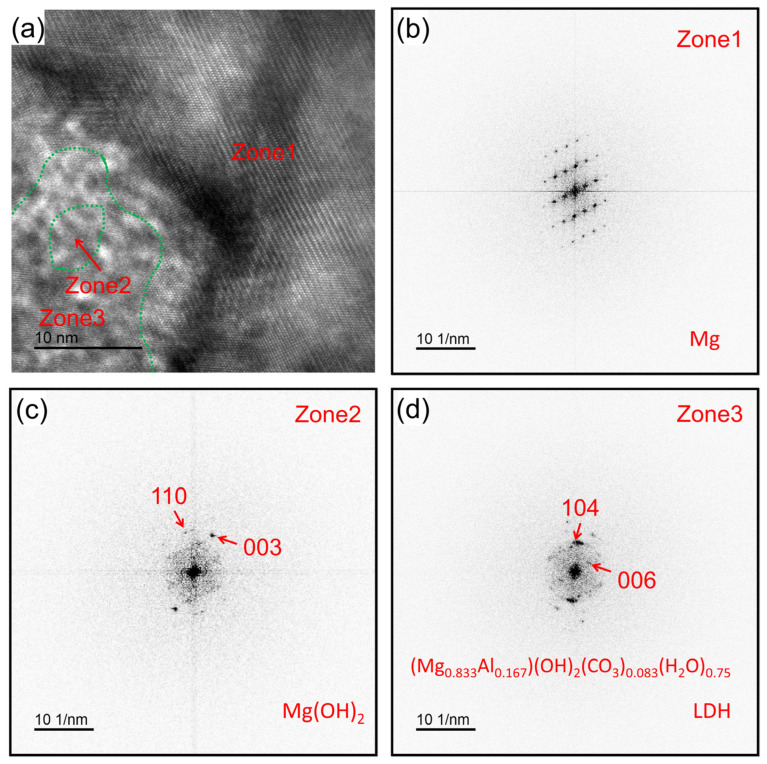
(**a**) High-resolution TEM of the corrosion front. (**b**–**d**) are representative NBED patterns of zone 1, zone 2 and zone 3 in (**a**), respectively.

The TEM sample was prepared using a focused ion beam (FIB), and Figure 3a depicts the selected area prior to the lift-out process. The white dashed line indicates the corrosion front. The TEM sample comprises both the degradation layer and the pristine section as indicated by the green dashed line, where the latter constitutes a minor fraction that amounts to less than 13% of the overall volume. The degradation layer is stratified into a hybrid layer (HL), an amorphous layer (AL), and a poor crystallinity layer (PCL) as depicted in Figure 3b,(c-1–c-6). The PCL layer exhibits a higher level of density compared to the AL layer. Figure 3(c-1–c-6) presents the elemental distribution map acquired via transmission electron microscopy (TEM). The corrosion front can be discerned independently based on the presence of Ca, P, O, and C elements. Notably, the Al exhibits a uniform distribution within the AL, which means that the Mg_17_Al_12_ is capable of undergoing complete degradation over a specific duration in vivo. Within the confines of the HL, the zones displaying pronounced aluminum enrichment coincide precisely with the crystalline Mg_17_Al_12_ phase. Both the AL layer and the PCL layer reveal an abundance of Ca and P elements. Yet, it is noteworthy that the concentration of these elements in the PCL layer surpasses that observed in the AL layer. The representative NBED patterns of HL, AL, and PCL are shown in Figure 3d, 3e, and 3f, respectively. The HL displays a thickness spanning between approximately 2 and 5 μm, with elegantly interwoven amorphous and crystalline Mg constituents. The thickness of the AL reaches up to 11 μm, and it is primarily composed of an amorphous structure. In the PCL, the amorphous phase poorly crystallizes in the bio-environments.

The morphology and elemental distribution of the HL layer are shown in Figure 4. The corrosion interface is approximately aligned parallel to the arrangement direction of the Mg_17_Al_12_, as depicted in Figure 4b,c. At a macroscopic level, corrosion is often impeded by the uniformly distributed precipitated phases. As shown in Figure 4d, Zn predominantly resides within the Mg_17_Al_12_. Based on the data presented in Figure 4c,e–h, it can be inferred that the main corrosion products in this layer are Mg/Ca phosphate. Micro-galvanic corrosion results in the formation of elongated corrosion channels within the alloy. These channels can effectively accommodate a significant volume of fluids while simultaneously facilitating the efficient transport of substances for the purpose of corrosion. By examining Figure 4a,b,g, it can be seen that a corrosion channel is entirely obstructed by magnesium carbonate. Within the Mg_17_Al_12_ of the non-corroded regions, the Mg–Al atomic ratio is 2.99, whereas in the Mg_17_Al_12_ of the HL layer, the atomic ratio is merely 0.47, as shown in Figure 4i. This implies that the Mg_17_Al_12_ is undergoing dealloying. The phenomenon of dealloying has also been observed in magnesium-calcium alloys [36].

Representative NBED patterns of HL, AL, and PCL layers are shown in Figure 5. It can be inferred that the HL layer contains both Mg/Ca phosphate crystals and non-crystalline regions, as seen in the diffraction patterns combined with the element mapping shown in Figure 4. From the corrosion interface and moving outwards, Mg/Ca phosphate progressively undergoes amorphization. In the AL layer, the Mg/Ca phosphate becomes entirely amorphous. Figure 5h is an exemplary representation from a series of continuous NBED patterns, demonstrating typical amorphous features. Figure 5i displays the diffraction pattern of the PCL layer, revealing the recrystallization of Ca/Al phosphate. As time goes on, the Mg in the Ca/Mg phosphate will be replaced by Ca.

In pursuit of a more insightful examination of the initial stage of the alloy corrosion within the body, a close-up high-resolution image captured in the vicinity of the corrosion front is shown in Figure 6. Three discernible regions are conspicuously evident, characterized by diffraction patterns: zone 1, zone 2, and zone 3. Zone 3 encapsulates Zone 2. These regions are demarcated by delineated green dashed lines. The corresponding rapid Fourier transformations for these distinct regions are visually depicted in Figure 6b–d. Zone 1, zone 2, and zone 3 represent distinct phases: Mg, Mg(OH)_2_, and (Mg_0.833_Al_0.167_)(OH)_2_(CO_3_)_0.083_(H_2_O)_0.75_ (layered double hydroxide, LDH). The LDH phase has been considered as a corrosion product of AZ series alloys exposed to actual atmospheric environments for a long time [37].

**Figure 7:**
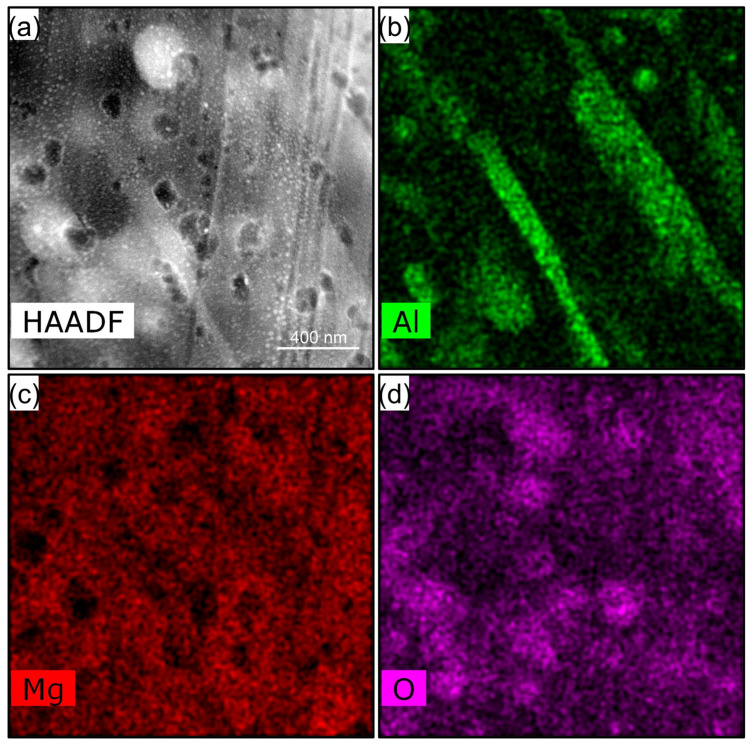
STEM-HAADF image (**a**) and the corresponding element maps (**b**–**d**) obtained from the hybrid layer.

The hybrid layer, along with its corresponding elemental distribution, is presented in Figure 7. The corrosion process of the alloy within the physiological environment initiates nanoscale pitting at localized sites according to the Mg and O element mapping. This pitting predominantly occurs at the magnesium phase along the interfaces between the matrix and the Mg_17_Al_12_. The Mg_17_Al_12_ remains intact. As the corrosion advances, these initial pits gradually enlarge, eventually coalescing to form an interconnected network.

## 4. Discussion

### 4.1. Structure and Composition of Degradation Layer

In the majority of studies, a common observation is the presence of duplex structures within the degradation layer [19,38,39]. These duplex structures typically consist of an outer compact layer and an inner porous layer. The outer compact layer is characterized by higher concentrations of calcium and phosphorus, suggesting that it is primarily composed of calcium phosphate compounds. In the outer layer, it appears that magnesium is nearly entirely replaced by calcium near the surface. The inner porous layer displays a uniform distribution with lower concentrations of calcium and phosphorus. In the current study, the obtained degradation inner layer can be more precisely categorized into two sub-layers: the outer main degradation layer and the inner hybrid-interface layer. In this research, we have employed TEM technology to delineate the degradation layer into three distinct layers from the outside to the inside, namely:

(1) The PCL layer: The amorphous phase poorly crystallizes in this layer. The main component of this layer is calcium phosphate. The kinetics of calcium phosphate formation on the surface are extensively elucidated in reference [40]. This layer possesses a relatively compact structure, which serves as an effective barrier to slow down the rate of substance exchange, thus establishing robust corrosion protection;

(2) The AL layer is primarily composed of amorphous Mg/Ca phosphates and exhibits a porous structure;

(3) The HL layer: This layer is composed of crystalline Mg(OH)_2_, LDH, amorphous magnesium phosphate, and magnesium carbonate. The fluid in certain localized areas of this layer is highly alkaline. The gaps formed after the degradation of the magnesium substrate are a highly efficient channel for mass transport.

### 4.2. Degradation Mechanism of AZ91 Alloy

Significant in vivo research has been conducted on the initial degradation of magnesium alloys after their implantation. The freshly exposed surface of the alloy promptly undergoes reactions with bodily fluids. As a consequence of electrochemical corrosion, the degradation proceeds continuously, resulting in the generation of oxygen-rich by-products such as MgO, Mg(OH)_2_, and Mg carbonates, or a combination of the three. Subsequent to this, the calcium and phosphorus gradually supplant the magnesium. The ion exchange between the degradation layer and the surrounding environment is hypothesized to occur concurrently with the mass transport phenomenon. This has been reported in the literature [38]. Amorphous phase poorly crystallizes and forms a PCL layer. When the substance transport is hindered by the PCL layer, the corrosion process tends to exhibit a milder character. The principal inetntion of this study is to comprehensively elucidate the degradation mechanisms at play during the stable degradation phase of magnesium alloys subsequent to the establishment of a surface protective layer.

**Figure 8:**
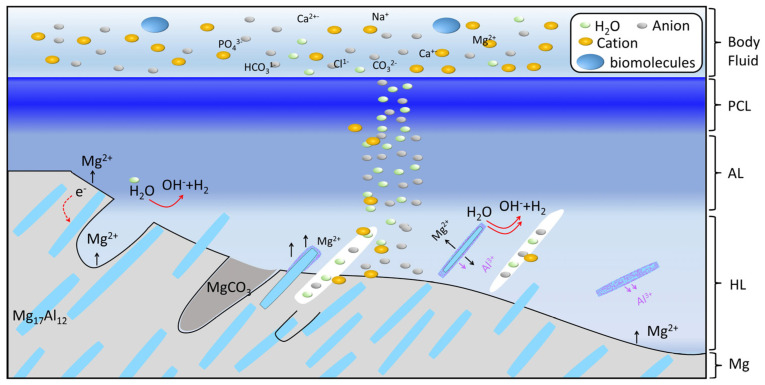
Schematic diagram of degradation channel mechanism of the AZ91 alloy membrane in vivo.

The steady-state degradation mechanism of AZ91 in vivo is illustrated in Figure 8. The galvanic coupling effect is the primary factor. On one hand, the open circuit potential of Mg_17_Al_12_ (~−1.05 V_Ag/AgCl_) in SBF solution is more positive than that of the Mg matrix (~−1.75 V_Ag/AgCl_) [41] in the alloy. The Mg_17_Al_12_ plays the part of a cathodal site as compared to the Mg matrix in solution over a range of pH values. The Mg is preferentially corroded and the Mg_17_Al_12_ is exposed. At the same time, corrosion channels also appear. Due to the cathodic protection effect caused by the corrosion of the magnesium matrix, this analysis shows that the phase corrosion is relatively slight, with only a few nanometers of protective film formed on the surface. This film turned out to be magnesium aluminum hydroxide [42]. On the other hand, the open circuit potential of Mg (−2.372 V_H^2^/H^+^_) has is more positive than that of Al (−1.66 V_H^2^/H^+^_) in the Mg_17_Al_12_ [43]. As for the precipitated phase in the free state, the magnesium and aluminum will also form galvanic corrosion. The magnesium will dissolve preferentially, along with a small amount of aluminum. As the reaction proceeds, the precipitated phase will gradually turn into a full Al phase, and eventually the full Al phase will also gradually dissolve into Al^3+^ ions in body fluids.

The main cations are Mg^2+^, Al^3+^, and Ca^2+^. The anions mainly comprise OH^−^ from the half reaction, 2H_2_O+2e^−^ → 2OH^−^+H_2_, and other anions from the body fluid, including Cl^−^, CO_3_^2−^, PO_4_^3−^, H_2_PO_4_^−^, HPO_4_^2−^, and HCO_3_^−^, in the HL layer at the steady-state degradation stage. H_2_PO_4_^−^, HPO_4_^2−^, and HCO_3_^−^ would react with OH^−^ to produce CO_3_^2−^, PO_4_^3−^ at the corrosion front. Mg^2+^, Al^3+^, CO_3_^2−^, and OH^−^ can produce Mg(OH)_2_ and LDH. LDH acts as a barrier against the body fluid attack. However, the Cl^−^ can insert into the LDH to accelerate its decomposition over the long term. The solubility product constants (Ksp) for Mg_3_(PO_4_)_2_ and Ca_3_(PO_4_)_2_ are 1 × 10^−32^ and 2.0 × 10^−29^, which are much smaller than those of Mg(OH)_2_ (8.9 × 10^−12^) and Ca(OH)_2_ (6.5 × 10^−6^) [44]. Ma/Ca phosphate, as the corrosion barrier, precipitates first [8]. Therefore, the precipitate corrosion layer in the HA layer is mainly Ma/Ca phosphate. These Ma/Ca phosphates are amorphous nanoparticles and connect with each other to form a porous and loose AL layer. If the carbonate concentration is high enough, magnesium carbonate can also form. The magnesium carbonate is meta-stable, since its K_SP_ is 1 × 10^−5^ [44]. The magnesium carbonate in the HL will convert to Ma/Ca phosphate in AL. And the poor crystallization of the amorphous Ma/Ca phosphates occurs through a complex method such as particle attachment in PCL layer. After a longer period, the Mg in the Mg/Ca phosphate can be replaced by Ca, forming a more stable crystalline structure.

The AZ91 corroded slowly in vivo at the steady-state degradation stage because, firstly, the compact PCL layer effectively blocks mass transport and, secondly, a continuous β-Mg_17_Al_12_ phase acts as a corrosion barrier that blocks the propagation of a corrosion front, although it serves as a cathode in micro-galvanic corrosion. Thirdly, the LDH has a shielding effect, especially for Cl^−^.

Although we have discussed the degradation mechanisms of AZ91 during the stable degradation stage in vivo, it is important to acknowledge that these findings may not fully capture the complexity of the early or transition stages of degradation. The stable stage represents only one aspect of the overall degradation process, and further investigation is needed to understand the complete timeline and influencing factors. The complexity of their degradation behavior in physiological environments remains a critical challenge in the development and application of magnesium-based biomaterials. This complexity arises from the dynamic interplay of multiple factors that influence degradation, including local pH variations, mechanical stresses, biological interactions, and fluid dynamics [45]. For example, pH shifts due to inflammatory responses can accelerate or decelerate corrosion rates [24], while mechanical loading may induce localized stress corrosion cracking. Additionally, protein adsorption [10], immune cell activity, and tissue integration can modulate degradation pathways in unpredictable ways. Moreover, the heterogeneous nature of physiological environments—such as differences in blood flow [46], interstitial fluid circulation, and ion concentrations across tissues—adds further complexity.

## 5. Conclusions

This study investigates the degradation mechanism of AZ91 magnesium alloy in a critical-size rat defect model, focusing on the characteristics of the corrosion layer over an 8-week period in vivo. The alloy maintains a stable and low degradation rate during this period. The key findings of this study are as follows:

1. The degradation layer consists of three distinct sub-layers: the PCL layer, the AL layer, and the HL layer. The PCL layer exhibits a relatively dense and compact structure, while the AL layer is characterized by a loose and porous morphology. The HL layer, in contrast, contains degradation channels;

2. The primary components of each layer are as follows: the PCL layer primarily contains calcium phosphate, the AL layer is composed of magnesium/calcium phosphate, and the HL layer is composed of magnesium/calcium phosphate, layered double hydroxide (LDH), and magnesium hydroxide;

3. The corrosion resistance of AZ91 in vivo is effectively enhanced by the presence of LDH in the HL layer, calcium in the phosphate-rich PCL layer, and the uniformly distributed Mg_17_Al_12_ phase, which collectively contribute to slowing the degradation rate.

## Figures and Tables

**Table 1:** Chemical compositions (wt. %) of the 5 AZ91 alloy samples measured by inductively coupled plasma-mass spectrometry.

Element	Al	Zn	Mn	Ce	Si	Cu	Fe	Ni	Mg
Content	9.03 ± 0.01	0.50 ± 0.01	0.27 ± 0.01	0.019 ± 0.002	<0.01	<0.01	<0.01	<0.01	Bal.

## Data Availability

The raw data supporting the conclusions of this article will be made available by the authors on request.
